# Environment influences the genetic structure and genetic differentiation of *Sassafras tzumu* (Lauraceae)

**DOI:** 10.1186/s12862-024-02264-9

**Published:** 2024-06-13

**Authors:** Bicai Guan, Qian Liu, Xiang Liu, Xi Gong

**Affiliations:** School of Life Sciences, Nanchang University, Nanchang, 330031 China

**Keywords:** *Sassafras tzumu*, Landscape genetics, Environmental distance, Genetic diversity, Genetic differentiation

## Abstract

**Background:**

*Sassafras tzumu*, an elegant deciduous arboreal species, belongs to the esteemed genus *Sassafras* within the distinguished family Lauraceae. With its immense commercial value, escalating market demands and unforeseen human activities within its natural habitat have emerged as new threats to *S. tzumu* in recent decades, so it is necessary to study its genetic diversity and influencing factors, to propose correlative conservation strategies.

**Results:**

By utilizing genotyping-by-sequence (GBS) technology, we acquired a comprehensive database of single nucleotide polymorphisms (SNPs) from a cohort of 106 individuals sourced from 13 diverse *Sassafras tzumu* natural populations, scattered across various Chinese mountainous regions. Through our meticulous analysis, we aimed to unravel the intricate genetic diversity and structure within these *S. tzumu* populations, while simultaneously investigating the various factors that potentially shape genetic distance. Our preliminary findings unveiled a moderate level of genetic differentiation (*F*_ST_ = 0.103, *p* < 0.01), accompanied by a reasonably high genetic diversity among the *S. tzumu* populations. Encouragingly, our principal component analysis painted a vivid picture of two distinct genetic and geographical regions across China, where gene flow appeared to be somewhat restricted. Furthermore, employing the sophisticated multiple matrix regression with randomization (MMRR) analysis method, we successfully ascertained that environmental distance exerted a more pronounced impact on genetic distance when compared to geographical distance (*β*_E_ = 0.46, *p* < 0.01; *β*_D_ = 0.16, *p* < 0.01). This intriguing discovery underscores the potential significance of environmental factors in shaping the genetic landscape of *S. tzumu* populations.

**Conclusions:**

The genetic variance among populations of *S. tzumu* in our investigation exhibited a moderate degree of differentiation, alongside a heightened level of genetic diversity. The environmental distance of *S. tzumu* had a greater impact on its genetic diversity than geographical distance. It is of utmost significance to formulate and implement meticulous management and conservation strategies to safeguard the invaluable genetic resources of *S. tzumu*.

**Supplementary Information:**

The online version contains supplementary material available at 10.1186/s12862-024-02264-9.

## Introduction

The genetic architecture and genetic variation serve as the underpinnings for plants to acclimate to their surroundings and ensure their survival and reproductive success [1, 2]. The greater the wealth of genetic diversity, the more adaptable a species becomes in navigating its environment, thus bestowing it with a heightened advantage in terms of survival and evolutionary potential [3]. Factors such as the geographical distribution range, growth patterns, and reproductive strategies exert influence on the genetic structure and diversity of plant species [4]. Species with a broader range of distribution may exhibit a greater tapestry of genetic variability [5]. Self-pollinating species, due to reproductive assurance, possess a more extensive geographical reach compared to their hybrid counterparts [6–9]. The duration of a species' generational growth profoundly impacts its genetic diversity. Species with shorter generations are anticipated to possess more restricted gene pools, hence fostering population isolation. Conversely, species with lengthier generation times are expected to experience a more gradual decline in genetic diversity [10], albeit this trend could also lead to genetic homogeneity. Furthermore, species with limited dispersal capabilities tend to exhibit greater differentiation, while those with long-distance dispersal mechanisms are more prone to population homogenization [10]. Thus, the genetic structure and diversity of species can, to a certain extent, serve as a reflection of their genetic evolutionary potential and adaptive capacity to their environment [11].

*Sassafras tzumu*, an arboreal species, pertains to the botanical family Lauraceae and falls under the genus *Sassafras*. It thrives abundantly across the southern region of the Yangtze River in China, flourishing at altitudes ranging from 100 to 1900 m amidst both sparse and dense woodland habitats [12]. Although the International Union for Conservation of Nature (IUCN) classifies *S. tzumu* as least concern (LC) and this species is not listed in the China Species Red List, in 2017, *S. tzumu* was included in the Reference List of Major Cultivated Precious Tree Species in China by the State Forestry Administration [13]. It usually begins to bloom from an early spring. With its vibrant crimson foliage in the autumn, ever-changing leaf patterns, and exceptional quality, *S. tzumu* not only served as a splendid ornamental tree but also boasted remarkable timber properties [14]. Moreover, the roots and bark possessed medicinal properties, known for their ability to enhance blood circulation, disperse blood stasis, alleviate wind and dampness, and treat contusions and lumbar muscle strains [13]. Regrettably, due to its immense commercial value, certain regions had suffered from rampant deforestation (http://qjwb.thehour.cn/html/2017-07/18/content_3547987.htm?div=-1) and theft of *S. tzumu* specimens (http://www.jiande.gov.cn/art/2018/6/27/art_1468690_18903167.html). In recent decades, escalating market demands and unforeseen human activities within its natural habitat had emerged as new threats to *S. tzumu* [15].

Presently, investigations pertaining to the genetic diversity and genetic structure of *S. tzumu* populations predominantly center around the analysis of genetic diversity utilizing isoenzymes and the development of microsatellite loci [14, 16–18]. Wang et al. [17] used 27 polymorphic nSSRs of *S. tzumu* to conduct genetic diversity, genetic structure analysis and mantel tests, thus providing corresponding theoretical basis for the protection and utilization of *S. tzumu*. A study conducted an analysis of the genetic structure of three diminutive natural populations of *S. tzumu* in Hubei Province, wherein the findings explicated that the proportion of polymorphic loci in the three populations stood at 49.5%. These populations were characterized by an inadequacy of heterozygotes and a surplus of homozygotes, thus signifying a state of disequilibrium [18]. Jiang et al. [14] found that five wild populations of *S. tzumu* at different altitudes had high genetic diversity (*H*_E_ = 0.84) and moderate genetic differentiation with 13 polymorphic SSR markers from *S. randaiense* and *Cinnamomum camphora.* Employing ArcGIS 10.2 and MaxEnt 3.3.2, the distribution pattern of *S. tzumu* in different timeframes was simulated. The outcomes demonstrated that precipitation of driest month, precipitation of wettest month, temperature seasonality, and mean temperature of wettest quarter chiefly influenced the distribution. Furthermore, a comparison between the simulated outcomes of the distribution range during distinct periods revealed that the suitable habitat for *S. tzumu* contracted and shifted from the southern to the northern regions [19].

Genotyping-by-sequence (GBS) technology represented one of the streamlined genome sequencing methodologies, aiming to reduce genome complexity through the application of restriction enzymes and the incorporation of single nucleotide polymorphisms (SNPs) [20]. This technique yielded a substantial number of SNPs, which were harnessed for exploring interspecies diversity, constructing haplotype maps, conducting genome-wide association studies, and facilitating genome selection [21]. Notably, GBS offered the advantage of requiring fewer steps for database construction and enabled the establishment of databases for a large number of samples [22]. In certain genomic studies, the utilization of genotyping-by-sequencing (GBS) technology allowed for the elucidation of species' genetic diversity and hybridization characteristics [23].

The fragmentation of habitats caused by anthropogenic activities and other contributing factors, coupled with the escalating effects of global climate change, has resulted in a significant decline in the genetic diversity of numerous indigenous plant species. Such diminishment in genetic diversity has profound implications for the long-term viability and survival of these plants [24, 25]. In order to establish a robust theoretical framework for future judicious development and utilization of germplasm resource collections, this study employed cutting-edge genotyping-by-sequencing technology to delve into the intricacies of genetic diversity and genetic structure across thirteen distinct populations. The primary objectives of this research endeavor encompass the following: (1) elucidating the genetic diversity and genetic structure within the species *S. tzumu*; (2) discerning the interplay between genetic distance, geographical distance, and environmental factors; (3) formulating pertinent conservation policies. The findings of this study hold the potential to enhance our understanding of the genetic diversity and structural dynamics within the thirteen populations of *S. tzumu*, thereby furnishing the necessary scientific foundation and conservation strategies.

## Results

### Genotyping and sequencing analysis results

In this study, GBS technology was used to genotype 106 *S. tzumu* samples collected from 13 different distributions in China, and a total of 134,935 SNPs were obtained. A total of 11,862 SNPs were obtained after Vcftools filtering. Meanwhile, clean reads ranged from 2,946,429 (TMS10) to 7,999,731 (GZS03), and the mean value of clean reads of 106 samples was 4,693,475. HighQ reads ranged from 2,436,391 (TMS10) to 6,541,952 (JHS06), and the mean value was 3,815,536. HighQ reads rates ranged from 77% (SS01) to 88% (LS05), and the mean value of the HighQ reads rates was 83.22%. HighQ bases ranged from 345,967,522 (TMS10) to 928,957,184 (JHS06), and its mean value was 541,806,161. HighQ bases rates were from 73% (SS01) to 84% (LS05), and its average value was 78.77%. HighQ Q20 bases rates were from 98.934% (SS01) to 99.4015% (GZS08), HighQ Q30 bases rates were from 95.742% (GZS17) to 97.654% (GZS08), and their average values were 99.19% and 96.83%, respectively. HighQ N content ranged from 13 (FD08)—1738 (LS05), HighQ GC content ranged from 43.554 (WYS01) to 49.0725 (JGS02), and their mean values were 630.55 and 45.0424 (Supplementary file 1), respectively.

### Genetic diversity and genetic differentiation

The populations exhibited a number range of 2.020 (JGS) to 2.571 (SS) alleles (*N*_A_), with an average of 2.428. The effective number of alleles (*N*_E_) ranged from 1.912 (JGS) to 2.129 (SS), with an average of 2.057. The Shannon's information index (*I*) varied from 0.628 (JGS) to 0.779 (SS), with a mean of 0.734. Expected heterozygosity (*H*_E_) ranged from 0.426 (JGS) to 0.490 (SS), while observed heterozygosity (*H*_O_) ranged from 0.492 (JHS) to 0.595 (JGS). The average values of *H*_E_ and *H*_O_ for *S. tzumu* were 0.469 and 0.515, respectively. The polymorphism information content (*PIC*) ranged from 0.8396 (JGS) to 0.9676 (SS), with a mean of 0.9459 (Table 1). The results of AMOVA revealed that the genetic variation of different populations of *S. tzumu* was 10% (*p* < 0.01) among population, 19% (*p* < 0.01) among individual, and the primary variation in *S. tzumu* was attributed to differences within populations (71%, *p* < 0.01). Therefore, there was greater genetic differentiation within populations (Table 2). Moderate genetic differentiation was observed among the 13 populations of *S. tzumu* (*F*_ST_ = 0.103, *p* < 0.01). The inbreeding coefficient (*F*_IS_) was calculated to be 0.215 (*p* < 0.01). The number of migrants (*N*_m_) between the 13 populations exceeded 1 (*N*_m_ = 2.172; Table 3). This indicated that the 13 *S. tzumu* populations had more gene exchange overall at the species level.

**Table 1:** Genetic diversity index of the 13 populations of *Sassafras tzumu*

Population	*N*_A_	*N*_E_	*I*	*H*_E_	*H*_O_	*PIC*
FD	2.458	2.054	0.735	0.469	0.495	0.9459
GZS	2.486	2.069	0.745	0.473	0.502	0.9555
HS	2.425	2.071	0.742	0.474	0.522	0.9439
JGS	2.020	1.912	0.628	0.426	0.595	0.8396
JHS	2.447	2.043	0.729	0.466	0.492	0.9448
LCS	2.450	2.056	0.735	0.469	0.498	0.9446
LS	2.391	2.073	0.739	0.474	0.534	0.9363
ML	2.511	2.083	0.752	0.476	0.503	0.9559
MS	2.524	2.076	0.750	0.474	0.501	0.9610
SS	2.571	2.129	0.779	0.490	0.519	0.9676
TMS	2.506	2.081	0.751	0.476	0.505	0.9546
TTS	2.504	2.078	0.750	0.476	0.503	0.9566
WYS	2.274	2.019	0.703	0.458	0.532	0.9063
Mean	2.428	2.057	0.734	0.469	0.515	0.9459

*Abbreviations*: *N*_A_ Number of alleles, *N*_*E*_ Effective number of alleles, *I* Shannon's information index,* H*_O_ Observed heterozygosity, *H*_E_ Expected heterozygosity, *PIC* Polymorphism information content

**Table 2:** The results of analyses of molecular variance (AMOVA) for *Sassafras tzumu*

Source	df	SS	MS	Est. Var	Percentage of variation (%)	*P*-Value
Among Pops	12	46677.17	3889.764	145.62	10	< 0.01
Among Indiv	93	142978.7	1537.405	272.438	19	< 0.01
Within Pops	106	105208	992.528	992.528	71	< 0.01
Total	211	294863.8		1410.587	100	-

*Abbreviations*: *df* degrees of freedom, *SS* sums of squares, *MS* mean squares, *Est. Var.* estimated variance

**Table 3:** The change in genetic differentiation (*F*_ST_) (above diagonal) where *p* < 0.01, inbreeding coefficient (*F*_IS_), and number of migrants (*N*_m_) among 13 populations of *Sassafras tzumu*

*F*-Statistics	Value	*P*-Value
*F*_ST_	0.103	< 0.01
*F*_IS_	0.215	< 0.01
*N*_m_	2.172	

**Fig. 1:**
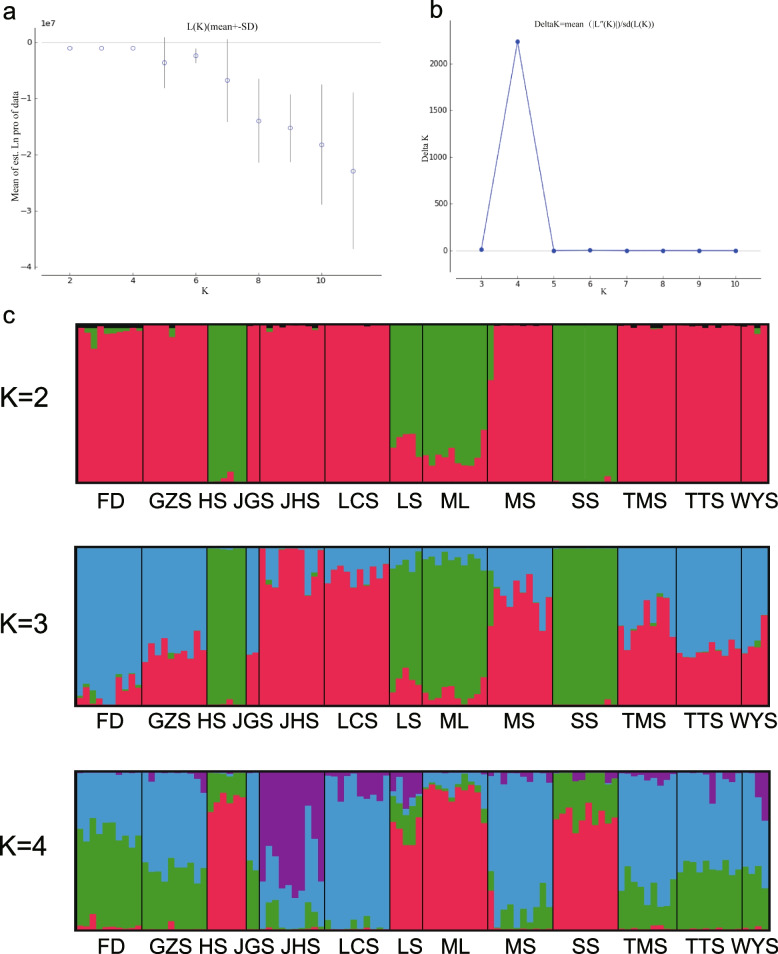
Genetic structure analyses of 13 populations of *Sassafras tzumu*. **a** Relationship between mean LnK and K value. **b** Relationship between Delta K and K value. **c** Genetic structure plots based on SNPs data (K = 2, 3 and 4)

Genetic structure analysis was conducted on the population data of 11,862 SNPs from the 13 populations. The results obtained from structure harvester indicated that the most suitable clustering was achieved when K = 4 (Fig. 1 a, b). When K = 2, 13 populations of *S. tzumu* could be divided into two genetic clusters, specifically, Hengshan Mountains (HS), Shaoshan Mountains (SS), Meiling Mountains (ML) and Lushan Mountains (LS) were divided into one cluster, and other populations were clustered into the other one. When K = 3, 13 populations of *S. tzumu* could be divided into three genetic clusters, in which HS, SS, ML and LS were still one cluster, Jiuhua Mountains (JHS), Longchi Mountains (LCS), Maoshan Mountains (MS) and Tianmu Mountains (TMS) were clustered into one cluster, Fuding Mountains (FD), Guanzhai Mountains (GZS), Jigong Mountains (JGS), Tiantai Mountains (TTS) and Wuyi Mountains (WYS) were clustered into one cluster. The findings of the genetic structure analyses revealed that the populations were organized into distinct clusters when K = 4. Cluster 1 consisted of samples from HS and SS, while cluster 2 comprised samples from ML and LS. Cluster 3 encompassed a significant number of samples from FD, GZS, JGS, MS, TMS, TTS and WYS. Additionally, a few specimens sourced from the JHS and LCS were consolidated within cluster 4 (Fig. 1c).

**Fig. 2:**
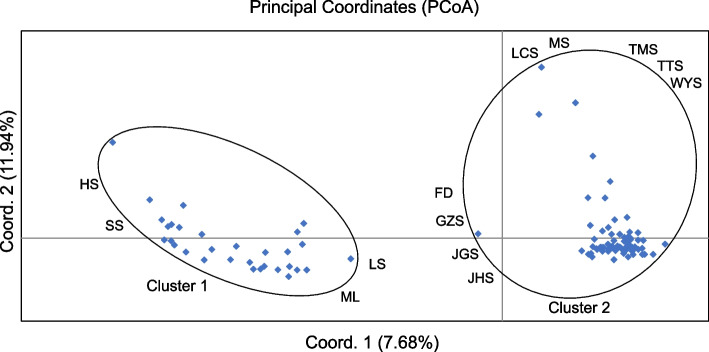
Principal component analyses (PCA) among 13 populations. PCA axis 1 explained 7.68% of the variance, whereas PCA axis 2 explained 11.94%. Different colors represented 13 populations

**Fig. 3:**
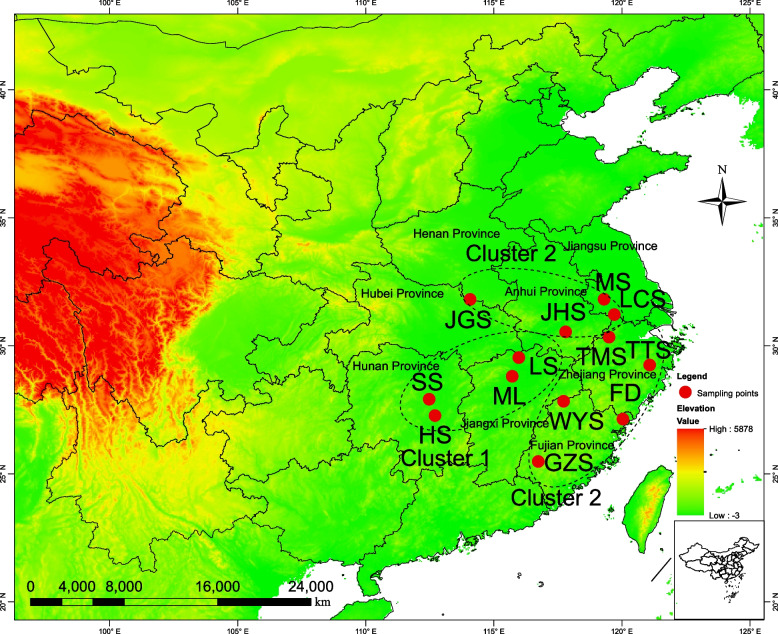
Red points were sampling locations of *S. tzumu* used in this study. The map was obtained from the National Geomatics Center of China (NGCC, https://www.ngcc.cn/ngcc/html/1/391/392/16114.html)

The first principal component (PC_1_) and the second principal component (PC_2_) of the PCA captured 7.68% and 11.94% of the total variance, respectively. The proportions of inertia associated with PC_1_ and PC_2_ were statistically significant (*p* < 0.01). The results showed that HS, SS, ML and LS clustered, and others segregated from them (Figs. 2 and 3).

### The multiple matrix regression with randomization (MMRR) analysis

**Fig. 4:**
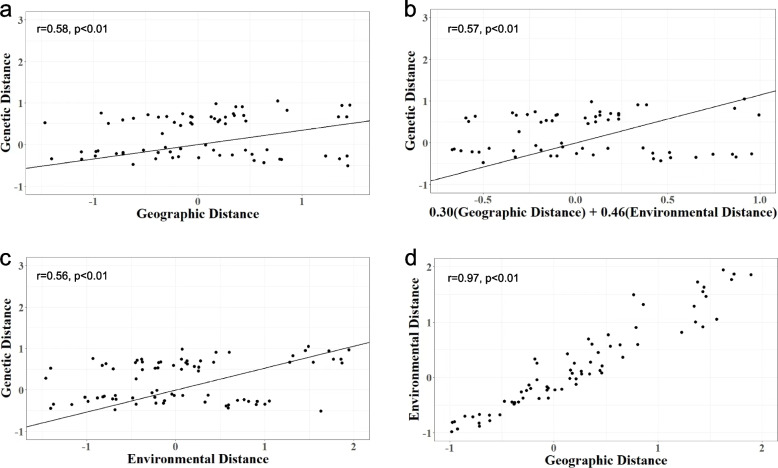
**a** Correlations between genetic distance and geographical distance. **b** Geographical distance and environmental distance effects on genetic distance. **c** Correlations between genetic distance and environmental distance. **d** Correlations between environmental distance and geographical distance

The utilization of MMRR analysis had unveiled a noteworthy positive correlation between genetic and geographical distances amongst the 13 populations (*r* = 0.58, *p* < 0.01; Fig. 4a). Within the 13 *S. tzumu* populations, the regression coefficient of environmental distance (*β*_E_ = 0.46, *p* < 0.01) exceeded that of geographical distance (*β*_D_ = 0.16, *p* < 0.01) by approximately threefold, implying that IBE predominantly accounted for the genetic distance. However, IBD also made a considerable contribution (Fig. 4b). Additionally, a significant association between genetic and environmental distances was observed (*r* = 0.56, *p* < 0.01; Fig. 4c). Furthermore, the outcomes of MMRR analyses demonstrated a significant association between environmental distances and geographical distances (*r* = 0.97, *p* < 0.01; Fig. 4d). In conclusion, the genetic distance among the 13 *S. tzumu* populations was jointly influenced by environmental and geographical distances (Table 4). Notably, environmental distance exhibited a more pronounced impact on genetic distance.

**Table 4:** The results of multiple matrix regression with randomization (MMRR) analyses of among genetic distance and different factors

File1	File2	*R*. Squared	*P*-Value
Gen	Geo	0.58	< 2.2e^−16^
Gen	Env	0.56	9.831e^−16^
Gen	Geo and Env	0.57	3.61e^−16^
Env	Geo	0.97	< 2e^−16^

*Abbreviations*: *Gen* genetic distance, *Geo* geographical distance, *Env* environmental distance

## Discussion

Genetic diversity is the main factor leading to population survival and evolution. It determines the coping ability of natural populations under the stress of various biotic and abiotic factors, which is crucial for the long-term survival of populations [26]. Genetic diversity is directly affected by species' own factors, including mating system, meiotic behavior, gene flow, mutation and natural selection [27–29]. In addition, human activities and environmental factors can also affect the level of genetic diversity of species, such as temperature, water, light, wind, soil salinity and nutrients, and the surrounding biological communities may exert different selective pressures on plant populations, thereby determining the evolution process of the population and shaping the genetic diversity and structure of populations [30, 31]. The greater the genetic variation of a species, the stronger its capacity to adapt to intricate and ever-changing environments [32]. We can quantify the genetic diversity of a species by calculating its expected heterozygosity (*H*_E_) [33]. The mean *H*_E_ value of the 13 *S. tzumu* populations stood at 0.469, surpassing that of 39 *Cinnamomum camphora* populations distributed in China (*H*_E_ = 0.322) [34]. Moreover, the *H*_E_ and *H*_O_ values of the 13 *S. tzumu* populations were lower than those of other arboreal species, such as *Xanthoceras sorbifolium* (*H*_E_ = 0.53 and *H*_O_ = 0.72) [35] and *Olea europaea* L. (*H*_E_ = 0.60 and *H*_O_ = 0.75) [36]. In contrast to the extensive genetic diversity observed in the aforementioned studies, it can be inferred that the level of genetic diversity of the *S. tzumu* populations examined in this study is inherently high.

Wright [37] believed that genetic differentiation coefficient (*F*_ST_) among populations was between 0 and 0.05, indicating that there was no genetic differentiation, and the *F*_ST_ value between 0.05 and 0.15 was moderately differentiated, then the *F*_ST_ value between 0.15 and 0.25 was highly differentiated. Furthermore, the mean *F*_ST_ value in our study was calculated to be 0.103, suggesting a moderate level of differentiation among 13 *S. tzumu* populations Compared with other Lauraceae plants, it was much lower than and *Cryptocarya chinensis* (*F*_ST_ = 0.141) [38] and *Litsea szemaois* (*G*_ST_ = 0.37) [39], and had a similar value with *Cinnamomun camphora* (*F*_ST_ = 0.109) [40]. The predominant distribution of genetic variation within 13 *S. tzumu* populations was observed to be within populations (71%, *p* < 0.01), whereas the genetic variation among populations was minimal (10%, *p* < 0.01). These findings aligned with the outcomes of previous studies on perennial plants [41] and further supported the notion that the genetic differentiation of *S. tzumu* primarily occurred within populations (93.6%, *p* < 0.001) [14]. Similar to Zhang's investigation, which focused on genus *Phoebe*, the primary source of variation in this species was within population (about 75.5%) rather than between populations (about 24.5%) [42]. Genetic diversity played a crucial role in adapting to extreme environments, and insufficient diversity could impede survival advantages in the face of environmental changes. Despite the current wide distribution of *S. tzumu*, it may lack sufficient genetic diversity to adapt to climate change. The inbreeding coefficient (*F*_IS_) was calculated to be 0.215. It was worth noting that a high inbreeding coefficient might result in a decrease in genetic diversity and an increase in genetic structure within the 13 *S. tzumu* populations [43].

The reproductive strategy of *S. tzumu* entails cross-pollination, facilitated by its amphimerotic flowers. Insects act as the primary agents for pollen dispersal, while birds play a role in fruit dispersion [44]. These two mechanisms of transmission are the key drivers of gene flow among populations. The transfer of genetic information occurs through the fruits, thereby contributing to gene flow [16]. This study revealed a certain degree of gene exchange (*N*m = 2.172) among the 13 *S. tzumu* populations. This could be attributed to the prolonged presence of frugivorous birds within the same population, who consumed the fruit seeds of *S. tzumu* and facilitated gene exchange within populations. Additionally, it was determined that the genetic differentiation level among the 13 natural *S. tzumu* populations was a moderate significance.

Based on the genetic structure analysis results of 13 populations of *S. tzumu*, *S. tzumu* could be divided into four clusters: cluster 1 was distributed in Hunan Province (HS and SS populations), cluster 2 was distributed in Jiangxi Province (ML and LS populations), cluster 3 (FD, GZS, JGS, MS, TMS, TTS and WYS) was mostly located in the eastern part of the populations, and cluster·4 included JHS and LCS populations (Fig. 1c). However, the results of PCA supported the HS, SS, ML and LS groups were cluster 1 and other groups were cluster 2 (Figs. 2 and 3). And the results of IBD analysis showed that the spatial distribution pattern of gene clusters and geographical distance of *S. tzumu* populations were extremely significant correlation (*r* = 0.58, *p* < 0.01, see Fig. 4a). Based on the study of major peaks and mountains in China by Wang et al. [45], the divergence of cluster 1 and cluster 2 was related to the geographical boundaries of Wuyi Mountains. The four populations in this study, HS, SS, ML and LS formed one cluster, which was consistent with cluster 2 in Zhou's research results [46]. A total of 9 populations of *S. tzumu*, FD, GZS, JGS, JHS, LCS, MS, TMS, TTS and WYS constituted another cluster, which was consistent with cluster 1 in Zhou's study. Finally, we are more supportive of the grouping of genetic structures in PCA.

Woody plants have a similar life history. *Tapiscia sinensis* (Tapisciaceae), whose life history is defined as outcrossing, and its seeds and pollen also spread over short distances [47]. In addition, other species with dioecious or morphologically dioecious but functionally dioecious breeding systems tend to maintain high genetic structure [48, 49]. Meanwhile, some potential factors may affect genetic structure, such as habitat fragmentation, breeding system and life forms of plants [50, 51].

The Multiple Matrix Regression with Randomization (MMRR) analysis is a linear regression model that aims to quantify the impact of multiple explanatory variables. A portion of the genetic variation could be accounted for geographical distance (*r* = 0.58, *p* < 0.01; Fig. 4a). The *S*. *tzumu* populations in cluster 1 and cluster 2 were distinct due to geographical distance. This could explain the results of the previous principal component analysis. Moreover, the results of MMRR analyses demonstrated that genetic distance was influenced by both geographical distance and environmental distance (*r* = 0.57, *p* < 0.01; Fig. 4b). But environmental distance of *S. tzumu* in this study had a greater and profounder impact on its genetic diversity than geographical distance (*β*_E_ = 0.46, *p* < 0.01; *β*_D_ = 0.16, *p* < 0.01; Fig. 4b), which could be attributed to its preference for warm and humid habitats based on its biological characteristics [52]. IBD and IBE (*β*_D_ = 0.404, *p* < 0.001; *β*_E_ = 0.382, *p* < 0.001) were the major and equal contributor to genetic differentiation among 61 populations of *Neolitsea sericea* (Lauraceae) in the study of Cao et al. [53]. Notably, IBE (*r* = 0.56, *p* < 0.01; Fig. 4c) exerted a significant influence on genetic distance. In the event of unfavorable environmental conditions, such as cold frosts or inclement weather during the pollination period, the pollination success of *S. tzumu* could be easily compromised [16], resulting in blooming without fruiting. Meanwhile, the distribution pattern of *S*. *tzumu* populations was found to be associated with elevation, slope direction, slope position, and humus thickness [54]. Research indicated that relative humidity (accounting for 26.2% of permutation importance), mean temperature of coldest quarter (16.6%), annual precipitation (12.6%), and temperature annual range (10.3%) were the primary factors influencing the distribution of *S. tzumu* in China [15].

In the research, 13 populations of *S. tzumu* exhibited a moderate degree of genetic diversity, rendering them less resilient to diverse habitats and intricate climate fluctuations, thereby posing a potential threat to the species' survival [55]. Consequently, it is imperative to implement protective measures. Firstly, there is a need to enhance the promotion of plant preservation and cultivate public consciousness regarding this matter. This can be achieved by implementing localized protective measures in economically underdeveloped and remote areas with limited transportation and inadequate awareness, as well as intensifying the dissemination of information regarding the conservation of *S. tzumu* to curb deforestation. Secondly, it is advisable to establish a nationwide network for ex situ conservation of plants, with National Botanical gardens playing a pivotal role. This network should encompass the creation of ecological corridors to facilitate gene flow among *S. tzumu* populations. Thirdly, in regions highly susceptible to climate change, it is recommended to introduce tree species that are known to adapt well to specific climatic conditions, instead of further expanding the cultivation of *S. tzumu*. Lastly, it is suggested to prioritize single plant selection and asexual propagation, while developing a comprehensive and scientifically guided breeding strategy for the development and utilization of *S. tzumu*.

In conclusion, we screened and developed 11,862 SNPs to effectively elucidate the genetic structure of *S. tzumu* populations. The findings revealed that *S. tzumu* populations exhibited a high level of genetic diversity. Simultaneously, it had come to our attention that the existing *S. tzumu* resources had suffered significant damage due to deforestation. In remote areas characterized by relatively underdeveloped economies, limited transportation, and insufficient protection and public awareness, it became imperative to implement in situ conservation measures and bolstered the dissemination of information regarding the protection of *S. tzumu* to deter indiscriminate deforestation. As for the development and utilization of *S. tzumu* resources, we propose placing emphasis on selective breeding of individual camphor trees, complemented by asexual reproduction. Additionally, it is crucial to formulate scientifical and comprehensive breeding strategies, manage *S. tzumu* genetic resources in a scientific manner, and enhance the sustainable utilization of these resources.

## Methods

### Collection and preservation of plant materials

During the summer of 2017, we gathered fresh leaves without pests or diseases as plant material samples from 13 naturally existing populations of *S. tzumu* across its range in China (Fig. 3). We made sure to collect individuals that were at least 50 m apart, with a collection of 2–10 individuals from each population. Ultimately, we carefully selected a total of 106 samples of *S. tzumu* for analysis (Table 5). To maintain the plant leaves' integrity, we carefully dried the leaves in sealed plastic bags with silica gel and stored them in a freezer at -20℃.

**Table 5:** The sampling locations of *Sassafras tzumu*

Code	Longitude	Latitude	Number of Samples	Temperature	Precipitation	Altitude (m)
FD	120.0398	27.13686	10	16.48	1686	497
GZS	116.7378	25.4839	10	19.03	1708	157
HS	112.7029	27.27414	6	16.03	1763	598
JGS	114.0766	31.81327	2	14.08	1212	158
JHS	117.8004	30.54553	10	16.12	1477	91
LCS	119.702	31.22193	10	15.52	1168	59
LS	115.9744	29.54243	5	12.58	1626	98
ML	115.7197	28.80984	10	17.04	898	397
MS	119.3055	31.8222	10	15.5	1078	384
SS	112.4749	27.91357	10	17.24	1510	479
TMS	119.5007	30.3415	9	14.1	1548	882
TTS	121.086	29.2522	10	12.44	1708	902
WYS	117.7218	27.83386	4	12.75	2135	957

### DNA extraction and genotyping-by-sequencing data set

For DNA extraction, dried floral leaves were subjected to the Plant Genomic DNA kit (Tiangen, Beijing, China) following the manufacturer's instructions. The extracted DNA from all samples was quantified using a Qubit spectrophotometer. To facilitate genotyping by sequencing, all samples were sent to the esteemed Institute of Shanghai OE Biotech. Co., Ltd. In brief, the DNAs from the 106 individuals were digested with *EcoR*I-HF and *Msp*I to reduce the complexity of the genome. Subsequently, a 106-plex GBS library, comprising 105 DNA samples and a negative control (no DNA), was meticulously prepared by ligating the digested DNA to unique barcode nucleotide adapters, followed by standard PCR amplification. Finally, the resulting 106-plex library was sequenced on a single lane of an HiSeq X Ten platform. Sequence data obtained from the Hiseq X Ten platform were processed for SNPs identification using the GBS pipeline integrated within Stacks 3.0.166 [56]. In brief, variants were filtered using Vcftools 0.1.14 [57] based on criteria such as a minimum depth of coverage (> 5), quality score (> 30), and an initial maximum missingness of 50% [58]. Subsequently, the dataset was examined to ensure that no samples exhibited high levels of missingness (all samples were found to have less than 30% missing data). The final set of SNPs was further filtered to retain those with a maximum of 20% missing values and a minor allele frequency below 0.05.

### Genetic diversity and genetic structure analysis

The resulting vcf file in our GBS data set was converted into compatible formats using PGDspider 2.0.9.0 [59] Genetic differentiation (*F*_ST_) was calculated for each SNP using GenAlEx 6.501 [60], and these values were utilized for analyses of molecular variance (AMOVA) among populations. To determine the number of genetic clusters across populations, the STRUCTURE 2.3.4 [61] was employed. The genetic structure analysis was conducted with three independent replicates, ranging from K = 2 to K = 13. Each run consisted of 5.0 × 10^4^ burn-in iterations and 3.0 × 10^4^ MCMC replications under the admixture model. The resulting output was submitted to the Structure Harvester [62] website for further analysis. The best K value was determined to represent the best number of clusters. The Q-matrix data, including Kx. indfile (individuals) and Kx. popfile (populations), obtained from multiple runs were downloaded. These data were then analyzed using CLUMPP [63] and visualized using DISTRUCT [64] and Adobe Illustrator 2021. To explore the clustering patterns between populations, principal components analysis (PCA) was performed using GenAlEx 6.501 [60].

### The multiple matrix regression with randomization analysis

Geographical distance and environmental distance affect gene flow and genetic differentiation between populations [65], and isolation by distance (IBD) and isolation by environment (IBE) are important ways to reflect that the complex landscapes affect the genetic structure of natural populations [66–70]. When gene flow may be affected by geographical and environmental variables, multiple matrix regression analysis (MMRR) [71] provides a valuable approach to quantify the impact of geographical and environmental isolation on genetic distance. In contrast to the many methods used to analyze distance matrices in landscape genetics [72–74], MMRR can ask not only whether variables are correlated but also how the dependent variable changes with respect to multiple independent variables, contributing to a comprehensive understanding of how landscape influences gene flow patterns.

In the study, the geosphere package in R software [75] was utilized to compute the euclidean distance of the latitude and longitude information from 13 populations. Additionally, MEGA 11 [76] was employed to determine the genetic distance among these populations. In this study, we used climate variables, soil pH and elevation as environmental factors. The climate variables (1970–2000) and elevation layer were downloaded from WorldClim website (https://worldclim.org), and soil pH was downloaded from ISRIC — World Soil Information (https://www.isric.org/). Then, we used Maxent 3.3.1 [77] to simulate the suitable layer in the species distribution model. SDM Tools in ArcMap 10.8 was used for converting from environment suitability layer to migration resistance layer. The value of the environmental layer in sampling locations was extracted from the matrix format utilizing ArcMap 10.8 [78], and the specifical operation was “Spatial Analyst Tools—Extract by points”.

The isolation by distance (IBD) and isolation by environment (IBE) were quantified using the "MMRR" function in R software [79]. We used genetic information as the response variable, with geographical and environmental distances serving as explanatory variables to describe the patterns of IBD and IBE, the correlation between the three matrices was computed to assess the impact of geographical distance and environmental distance on genetic distance.

### Supplementary Information

Supplementary Material 1.

## Data Availability

The datasets generated and/or analyzed during the current study are available in the Figshare repository under persistent web link “https://figshare.com/articles/dataset/Data_in_VCF_format_for_single_nucleotide_polymorphisms_SNPs_of_13_i_Sassafras_tzumu_i_Lauraceae_populations_with_106_individuals/24899328”.
