# Supplementary material for: Environment influences the genetic structure and genetic differentiation of Sassafras tzumu (Lauraceae)
Source: BMC Ecol Evol. 2024 Jun 13;24:80. doi: 10.1186/s12862-024-02264-9 (PMC11170782; doi:10.1186/s12862-024-02264-9)
Supplement: Supplementary file 1 — Supplementary Material 1. [file 12862_2024_2264_MOESM1_ESM.docx]

**Table 1** GBS data statistics table.

| Sample | Clean Reads | HighQ Reads | HighQ Reads Rate (%) | HighQ Bases (bp) | HighQ Bases Rate (%) | HighQ Q20 Bases Rates (%) | HighQ Q30 Bases Rates (%) | HighQ N Content | HighQ GC Content |
| --- | --- | --- | --- | --- | --- | --- | --- | --- | --- |
| JHS15 | 3,882,314 | 2,962,604 | 78.92 | 420,689,768 | 74.71 | 99.1885 | 96.771 | 228 | 44.6 |
| JHS08 | 3,733,597 | 2,869,130 | 79.07 | 407,416,460 | 74.85 | 99.162 | 96.682 | 271 | 45.0595 |
| FD06 | 4,010,467 | 3,080,513 | 79.49 | 437,432,846 | 75.25 | 99.188 | 96.7715 | 277 | 44.982 |
| FD11 | 3,693,953 | 2,952,413 | 80.72 | 419,242,646 | 76.42 | 99.2745 | 97.0755 | 260 | 44.792 |
| TMS11 | 4,856,382 | 3,847,865 | 80.53 | 546,396,830 | 76.23 | 99.26 | 97.0225 | 350 | 44.988 |
| JHS13 | 3,827,880 | 2,984,178 | 79.11 | 423,753,276 | 74.89 | 99.1535 | 96.6525 | 258 | 45.1235 |
| HS06 | 4,723,429 | 3,940,814 | 86.36 | 559,595,588 | 81.76 | 99.3065 | 97.3175 | 953 | 44.795 |
| TTS17 | 3,238,031 | 2,692,031 | 86.50 | 382,268,402 | 81.89 | 99.363 | 97.5345 | 762 | 44.79 |
| JHS04 | 3,597,326 | 2,871,653 | 81.53 | 407,774,726 | 77.19 | 99.2465 | 96.969 | 327 | 45.369 |
| ML02 | 3,815,202 | 2,956,368 | 79.41 | 419,804,256 | 75.18 | 99.1435 | 96.5835 | 254 | 44.514 |
| LCS09 | 4,084,420 | 3,415,979 | 86.53 | 485,069,018 | 81.91 | 99.3525 | 97.5105 | 906 | 44.7155 |
| ML05 | 3,748,276 | 3,114,379 | 85.88 | 442,241,818 | 81.30 | 99.3055 | 97.3215 | 787 | 44.091 |
| JHS10 | 3,708,441 | 2,961,760 | 81.45 | 420,569,920 | 77.10 | 99.224 | 96.8825 | 247 | 44.5925 |
| JHS11 | 6,222,974 | 5,139,289 | 85.60 | 729,779,038 | 81.03 | 99.282 | 97.2365 | 1441 | 45.361 |
| GZS13 | 3,545,266 | 2,695,768 | 81.82 | 382,799,056 | 77.46 | 99.2235 | 96.807 | 209 | 49.0725 |
| MS03 | 4,243,878 | 3,350,052 | 80.07 | 475,707,384 | 75.80 | 99.1515 | 96.6435 | 257 | 44.7045 |
| TMS05 | 3,917,641 | 3,094,213 | 80.90 | 439,378,246 | 76.59 | 99.2155 | 96.867 | 254 | 44.451 |
| HS01 | 7,150,666 | 6,126,925 | 86.97 | 870,023,350 | 82.33 | 99.315 | 97.3235 | 1671 | 47.2405 |
| MS13 | 4,932,163 | 3,827,293 | 79.58 | 543,475,606 | 75.34 | 99.1325 | 96.582 | 281 | 45.299 |
| LCS05 | 6,098,662 | 4,934,221 | 82.38 | 700,659,382 | 77.98 | 99.2735 | 97.064 | 418 | 44.2335 |
| GZS08 | 3,891,279 | 3,305,214 | 87.59 | 469,340,388 | 82.92 | 99.4015 | 97.654 | 757 | 46.761 |
| TMS10 | 2,946,429 | 2,436,391 | 85.92 | 345,967,522 | 81.34 | 99.271 | 97.1935 | 701 | 44.2095 |
| LCS15 | 4,941,951 | 3,944,350 | 82.20 | 560,097,700 | 77.82 | 99.2025 | 96.8185 | 292 | 44.3915 |
| WYS03 | 3,421,612 | 2,881,603 | 85.88 | 409,187,626 | 81.30 | 99.288 | 97.2505 | 492 | 44.9165 |
| JHS18 | 3,763,818 | 3,100,978 | 85.57 | 440,338,876 | 81.00 | 99.284 | 97.252 | 644 | 44.753 |
| WYS02 | 3,723,025 | 3,084,163 | 86.14 | 437,951,146 | 81.54 | 99.341 | 97.4675 | 670 | 44.951 |
| GZS04 | 4,375,837 | 3,623,539 | 86.12 | 514,542,538 | 81.53 | 99.3265 | 97.39 | 561 | 44.8605 |
| MS07 | 4,210,036 | 3,370,890 | 81.61 | 478,666,380 | 77.25 | 99.2505 | 97.001 | 290 | 44.228 |
| HS04 | 3,631,760 | 3,025,501 | 86.57 | 429,621,142 | 81.95 | 99.3165 | 97.35 | 502 | 44.8145 |
| LCS08 | 5,071,931 | 4,030,477 | 80.64 | 572,327,734 | 76.34 | 99.1245 | 96.5345 | 211 | 44.065 |
| SS03 | 5,534,619 | 4,517,319 | 85.32 | 641,459,298 | 80.77 | 99.272 | 97.18 | 766 | 44.559 |
| LCS18 | 5,735,097 | 4,557,079 | 82.03 | 647,105,218 | 77.66 | 99.2455 | 96.966 | 365 | 44.1165 |
| TTS12 | 5,505,686 | 4,674,268 | 86.68 | 663,746,056 | 82.06 | 99.33 | 97.4035 | 575 | 44.1855 |
| LCS04 | 3,430,744 | 2,634,362 | 78.14 | 374,079,404 | 73.97 | 98.956 | 95.818 | 281 | 44.185 |
| FD02 | 3,477,590 | 2,775,082 | 80.44 | 394,061,644 | 76.15 | 99.163 | 96.598 | 284 | 45.433 |
| GZS07 | 3,490,284 | 2,940,610 | 86.01 | 417,566,620 | 81.42 | 99.197 | 96.92 | 812 | 46.071 |
| MS09 | 3,544,771 | 2,673,766 | 77.85 | 379,674,772 | 73.7 | 98.958 | 95.866 | 209 | 45.315 |
| ML03 | 3,547,798 | 3,040,108 | 87.42 | 431,695,336 | 82.76 | 99.313 | 97.401 | 1017 | 43.554 |
| SS15 | 3,577,202 | 2,838,746 | 80.4 | 403,101,932 | 76.11 | 99.161 | 96.54 | 304 | 45.565 |
| ML14 | 3,583,744 | 2,710,465 | 77.95 | 384,886,030 | 73.8 | 98.967 | 95.884 | 227 | 44.43 |
| MS06 | 3,593,153 | 2,774,176 | 78.31 | 393,932,992 | 74.13 | 98.967 | 95.873 | 246 | 45.04 |
| FD18 | 7,424,836 | 5,842,919 | 79.963 | 829,694,498 | 74.497 | 99.0715 | 96.255 | 626 | 44.9255 |
| JHS09 | 3,724,331 | 3,176,457 | 87.14 | 451,056,894 | 82.49 | 99.303 | 97.372 | 1177 | 44.544 |
| SS16 | 3,748,189 | 3,088,471 | 85.78 | 438,562,882 | 81.21 | 99.202 | 96.965 | 986 | 44.507 |
| LS02 | 3,755,405 | 3,145,215 | 86.37 | 446,620,530 | 81.76 | 99.219 | 97.044 | 988 | 44.843 |
| HS03 | 3,758,219 | 3,246,164 | 87.33 | 460,955,288 | 82.67 | 99.314 | 97.403 | 1185 | 44.828 |
| TMS01 | 3,814,950 | 3,279,674 | 87.47 | 465,713,708 | 82.8 | 99.317 | 97.431 | 1154 | 43.989 |
| GZS18 | 3,854,299 | 2,935,626 | 78.94 | 416,858,892 | 74.73 | 99.058 | 96.121 | 285 | 46.501 |
| HS02 | 3,896,386 | 3,245,793 | 86.06 | 460,902,606 | 81.47 | 99.227 | 97.067 | 1146 | 44.577 |
| GZS05 | 3,910,876 | 3,340,330 | 86.49 | 474,326,860 | 81.88 | 99.295 | 97.306 | 1056 | 44.698 |
| SS10 | 3,952,388 | 3,022,575 | 78.68 | 429,205,650 | 74.48 | 99.026 | 96.067 | 263 | 44.274 |
| TMS15 | 4,001,280 | 3,312,190 | 85.93 | 470,330,980 | 81.34 | 99.198 | 96.97 | 905 | 44.621 |
| MS02 | 4,026,051 | 3,079,771 | 78.61 | 437,327,482 | 74.42 | 98.993 | 96.01 | 258 | 45.047 |
| LCS02 | 4,269,397 | 3,308,990 | 79.61 | 469,876,580 | 75.36 | 99.071 | 96.245 | 320 | 45.442 |
| ML08 | 4,276,154 | 3,691,063 | 87.22 | 524,130,946 | 82.57 | 99.31 | 97.404 | 1224 | 43.72 |
| FD15 | 4,278,834 | 3,328,313 | 78.77 | 472,620,446 | 74.56 | 98.989 | 95.958 | 265 | 45.696 |
| SS01 | 4,293,530 | 3,272,089 | 77.3 | 464,636,638 | 73.18 | 98.934 | 95.749 | 284 | 44.677 |
| ML01 | 4,369,231 | 3,451,715 | 79.76 | 490,143,530 | 75.51 | 99.132 | 96.435 | 331 | 44.001 |
| WYS01 | 4,369,597 | 3,488,523 | 80.68 | 495,370,266 | 76.37 | 99.17 | 96.59 | 360 | 45.378 |
| TMS16 | 4,385,300 | 3,719,639 | 85.93 | 528,188,738 | 81.34 | 99.184 | 96.919 | 1261 | 45.487 |
| GZS17 | 4,436,656 | 3,387,248 | 77.39 | 480,989,216 | 73.26 | 98.939 | 95.742 | 277 | 44.403 |
| ML13 | 4,447,747 | 3,502,712 | 80.27 | 497,385,104 | 75.99 | 99.127 | 96.453 | 354 | 44.353 |
| MS08 | 4,559,682 | 3,755,678 | 85.86 | 533,306,276 | 81.28 | 99.201 | 96.977 | 1208 | 44.858 |
| LS04 | 4,584,023 | 3,867,107 | 86.69 | 549,129,194 | 82.07 | 99.248 | 97.139 | 1270 | 44.522 |
| TTS01 | 4,611,247 | 3,568,250 | 78.9 | 506,691,500 | 74.69 | 98.991 | 95.945 | 365 | 44.289 |
| MS12 | 4,620,612 | 3,839,532 | 86.08 | 545,213,544 | 81.49 | 99.221 | 97.047 | 1275 | 44.649 |
| LCS07 | 4,704,272 | 3,599,570 | 78.67 | 511,138,940 | 74.47 | 98.997 | 95.986 | 317 | 44.38 |
| SS08 | 4,731,457 | 4,019,787 | 86.64 | 570,809,754 | 82.02 | 99.213 | 97 | 1306 | 45.118 |
| FD05 | 4,816,959 | 3,693,136 | 77.58 | 524,425,312 | 73.44 | 98.951 | 95.825 | 370 | 44.387 |
| LS03 | 4,872,529 | 4,151,601 | 86.37 | 589,527,342 | 81.76 | 99.209 | 97 | 1318 | 44.652 |
| TMS13 | 4,896,533 | 3,784,483 | 78.36 | 537,396,586 | 74.18 | 98.968 | 95.862 | 326 | 46.182 |
| ML07 | 4,913,467 | 4,110,415 | 86.2 | 583,678,930 | 81.61 | 99.24 | 97.086 | 1230 | 44.405 |
| TMS02 | 4,923,455 | 4,173,952 | 86.62 | 592,701,184 | 82 | 99.207 | 96.993 | 1354 | 44.53 |
| SS04 | 5,066,237 | 4,360,644 | 87.08 | 619,211,448 | 82.43 | 99.311 | 97.39 | 1637 | 44.554 |
| LS01 | 5,104,311 | 4,395,145 | 88.11 | 624,110,590 | 83.41 | 99.328 | 97.466 | 1521 | 44.243 |
| SS09 | 5,132,425 | 4,355,656 | 87.48 | 618,503,152 | 82.81 | 99.313 | 97.398 | 1576 | 44.746 |
| JGS01 | 5,217,389 | 4,486,310 | 87.73 | 637,056,020 | 83.05 | 99.261 | 97.142 | 1274 | 48.185 |
| TTS20 | 5,324,932 | 4,359,728 | 85.31 | 619,081,376 | 80.76 | 99.183 | 96.894 | 1073 | 44.169 |
| SS18 | 5,489,775 | 4,636,750 | 85.8 | 658,418,500 | 81.22 | 99.188 | 96.922 | 1256 | 44.741 |
| LS05 | 5,665,832 | 4,912,001 | 88.25 | 697,504,142 | 83.54 | 99.341 | 97.506 | 1738 | 44.244 |
| MS11 | 5,743,731 | 4,866,754 | 86.15 | 691,079,068 | 81.55 | 99.189 | 96.934 | 1484 | 45.141 |
| GZS02 | 5,750,673 | 4,836,778 | 85.49 | 686,822,476 | 80.93 | 99.179 | 96.862 | 1357 | 45.379 |
| SS19 | 5,936,024 | 4,969,998 | 85.89 | 705,739,716 | 81.3 | 99.221 | 97.034 | 1606 | 44.528 |
| ML06 | 6,006,987 | 5,099,375 | 85.96 | 724,111,250 | 81.38 | 99.192 | 96.931 | 1524 | 43.848 |
| TMS14 | 6,014,145 | 5,116,210 | 87.36 | 726,501,820 | 82.7 | 99.303 | 97.377 | 1727 | 44.69 |
| ML12 | 6,017,212 | 5,125,533 | 86.62 | 727,825,686 | 82 | 99.215 | 97.003 | 1511 | 43.837 |
| JGS02 | 6,608,302 | 5,100,905 | 79.62 | 724,328,510 | 75.37 | 99.063 | 96.223 | 528 | 47.09 |
| GZS03 | 7,999,731 | 6,258,547 | 80.15 | 888,713,674 | 75.88 | 99.113 | 96.38 | 676 | 43.719 |
| TTS13 | 3,504,925 | 2,807,924 | 82.01 | 398,725,208 | 77.63 | 99.115 | 96.529 | 39 | 46.567 |
| TTS04 | 3,668,390 | 3,014,949 | 83.91 | 428,122,758 | 79.44 | 99.261 | 97.072 | 28 | 45.626 |
| FD08 | 3,732,428 | 2,989,013 | 83.48 | 424,439,846 | 79.03 | 99.207 | 96.916 | 13 | 46.51 |
| TTS10 | 4,101,138 | 3,331,497 | 84.05 | 473,072,574 | 79.57 | 99.234 | 97.031 | 36 | 45.193 |
| TTS09 | 4,012,152 | 3,209,207 | 81.51 | 455,707,394 | 77.16 | 99.105 | 96.514 | 20 | 45.611 |
| FD12 | 4,360,963 | 3,606,385 | 83.66 | 512,106,670 | 79.2 | 99.245 | 97.034 | 22 | 45.666 |
| FD04 | 4,394,982 | 3,481,341 | 80.9 | 494,350,422 | 76.58 | 99.077 | 96.421 | 21 | 46.612 |
| WYS04 | 4,710,395 | 3,724,323 | 81.87 | 528,853,866 | 77.5 | 99.108 | 96.497 | 60 | 47.728 |
| LCS20 | 5,464,004 | 4,370,933 | 82.79 | 620,672,486 | 78.38 | 99.15 | 96.703 | 42 | 45.218 |
| TTS11 | 5,801,126 | 4,803,574 | 84.51 | 682,107,508 | 80 | 99.283 | 97.16 | 66 | 45.222 |
| LCS16 | 4,965,104 | 4,087,372 | 84.06 | 580,406,824 | 79.57 | 99.256 | 97.057 | 32 | 45.493 |
| TTS07 | 6,355,551 | 5,040,427 | 82.95 | 715,740,634 | 78.53 | 99.161 | 96.757 | 40 | 45.444 |
| GZS01 | 5,996,222 | 4,736,632 | 80.72 | 672,601,744 | 76.41 | 99.067 | 96.396 | 62 | 47.398 |
| HS05 | 6,675,248 | 5,315,680 | 80.95 | 754,826,560 | 76.63 | 99.088 | 96.429 | 34 | 45.694 |
| FD09 | 6,909,930 | 5,615,517 | 83.62 | 797,403,414 | 79.16 | 99.209 | 96.941 | 41 | 45.776 |
| JHS16 | 7,610,398 | 6,010,658 | 81.7 | 853,513,436 | 77.34 | 99.117 | 96.604 | 26 | 45.262 |
| MS04 | 6,926,883 | 5,693,612 | 83.03 | 808,492,904 | 78.6 | 99.218 | 96.955 | 40 | 45.559 |
| JHS06 | 7,983,629 | 6,541,952 | 83.16 | 928,957,184 | 78.73 | 99.215 | 96.917 | 67 | 45.136 |
| Mean | 4,693,475 | 3,815,536 | 83.22 | 541,806,162 | 78.77 | 99.19 | 96.83 | 630.55 | 45.0424 |

**(**Sample: number of samples; Clean Reads: number of Clean Data Reads; HighQ Reads: number of high quality Reads; HighQ Reads Rate: ratio of high quality Reads to low N content Reads; HighQ Bases: Bases number of high-quality Reads; HighQ Bases Rate: Ratio of bases number of high-quality Reads to bases number of low-N content Reads; HighQ Q20 bases Rates: Proportion of high-quality Reads with base quality values greater than Q20; HighQ Q30 Bases Rates: Proportion of high-quality Reads with base quality values greater than Q30; HighQ N Content: Number of N bases in high-quality Reads; HighQ GC Content: GC content in high-quality Reads.**)**
